# The diagnostic value of ultrasonography in carpal tunnel syndrome: a comparison between diabetic and non-diabetic patients

**DOI:** 10.1186/1471-2377-13-65

**Published:** 2013-06-24

**Authors:** Nai-Wen Tsai, Lian-Hui Lee, Chi-Ren Huang, Wen-Neng Chang, Hung-Chen Wang, Yu-Jun Lin, Wei-Che Lin, Tsu-Kung Lin, Ben-Chung Cheng, Yu-Jih Su, Chia-Te Kung, Shu-Fang Chen, Cheng-Hsien Lu

**Affiliations:** 1 Department of Neurology, Chang Gung Memorial Hospital-Kaohsiung Medical Center, Chang Gung University College of Medicine, Kaohsiung, Taiwan; 2 Department of Neurosurgery, Chang Gung Memorial Hospital-Kaohsiung Medical Center, Chang Gung University College of Medicine, Kaohsiung, Taiwan; 3 Department of Biological Science, National Sun Yat-Sen University, Kaohsiung, Taiwan; 4 Department of Radiology, Chang Gung Memorial Hospital-Kaohsiung Medical Center, Chang Gung University College of Medicine, Kaohsiung, Taiwan; 5 Department of Medicine, Chang Gung Memorial Hospital-Kaohsiung Medical Center, Chang Gung University College of Medicine, Kaohsiung, Taiwan; 6 Department of Emergency Medicine, Chang Gung Memorial Hospital-Kaohsiung Medical Center, Chang Gung University College of Medicine, Kaohsiung, Taiwan

**Keywords:** Carpal tunnel syndrome, Cross-sectional area, Median nerve, Ultrasonography

## Abstract

**Background:**

To compare the value of ultrasonography for diagnosing carpal tunnel syndrome (CTS) in patients with and without diabetes mellitus (DM).

**Methods:**

Eighty non-DM and 40 DM patients with electromyography-confirmed CTS were assessed and underwent high-resolution ultrasonography of the wrists. Cross-sectional area (CSA) and flattening ratio (FR) of the median nerve were measured at the carpal tunnel outlet (D) and wrist crease (W).

**Results:**

The 80 non-DM and 40 DM patients had 81 and 59 CTS-hands, respectively. The CSA_D and CSA_W were significantly larger in the CTS-hands and DM-CTS-hands compared to the normal control (*p* < 0.001). However, there is no difference of CSA_D and CSA_W between DM and non-DM CTS patients. Receiver operating characteristics [ROC] curve analysis revealed that CSA_W ≥13 mm^2^ was the most powerful predictor of CTS in DM (area under curve [AUC] = 0.72; sensitivity 72.9%, specificity 61.9%) and non-DM (AUC = 0.72; sensitivity 78.5%, specificity 53.2%) patients. The CSA positively correlated with the distal motor latency of the median compound motor action potential (CMAP), distal sensory latency of the median sensory nerve action potential (SNAP), and latency of the median F wave, but negatively correlated with the amplitude of the median CMAP, amplitude of the median SNAP, and sensory NCV of the median nerve. Stepwise logistic regression revealed that CSA_W (OR 1.21, 95% CI 1.07-1.38; *p* = 0.003) was independently associated with CTS in DM patients and any 1 mm^2^ increase in CSA_W increased the rate of CTS by 28%.

**Conclusions:**

The CSA of the median nerve at the outlet and wrist crease are significantly larger in CTS hands in both DM and non-DM patients compared to normal hands. The CSA of the median nerve by ultrasonography may be a diagnostic tool for evaluating CTS in DM and non-DM patients.

## Background

Carpal tunnel syndrome (CTS) is the most common form of entrapment neuropathy and is characterized by symptoms associated with localized compression of the median nerve at the wrist [1]. The pathophysiology is not fully understood but two mechanisms are speculated. The early stage involves functional abnormalities due to the compression of the median nerve in the carpal tunnel, while the advanced stage involves structural abnormalities [2]. Diagnosis is based on clinical presentation and confirmed by electrodiagnostic studies [3], but nerve conduction studies (NCS) alone do not provide spatial information regarding the nerve or its attendant abnormalities.

Ultrasonography is a useful non-invasive diagnostic method for CTS because it is painless, easily accessible, and preferred by the patients [4,5]. There is considerable correlation between conduction abnormalities of the median nerve detected by electrodiagnostic tests and measurement of its cross-sectional area (CSA) by ultrasonography [6,7]. Most investigators agree that CSA measurement of the median nerve at the pisiform bone is a valid and reliable test for diagnosing CTS [8]. However, the standard criteria of CTS by ultrasonography are not well established, especially in diabetic patients suspected of CTS.

Although diabetes mellitus (DM) is a risk factor for CTS [9], reports about median nerve CSA measurements between CTS patients with and without DM are scant. The aim of this study was to evaluate whether or not ultrasonographic findings of the median nerve is different between DM and non-DM CTS patients. Particularly, this study was made to elucidate the diagnostic value of ultrasonography and correlate the diagnostic accuracy of ultrasonography with electrodiagnostic results in both DM and non-DM CTS patients.

## Methods

### Study participants

Patients with clinically suspicious CTS at the out-patient clinics of the Department of Neurology of Kaohsiung Chang Gung Memorial Hospital were evaluated. Eighty idiopathic CTS (non-DM) and 67 diabetic patients with clinical suspicion of CTS were initially included. After 27 with diabetic neuropathy were excluded, 40 DM patients were finally enrolled. The 80 non-DM participants had 81 hands with clinical symptoms and electrophysiologic diagnosis of CTS (“CTS-hands”), while the 40 DM patients had 59 hands that fulfilled the clinical and electrophysiologic diagnostic criteria of CTS (“DM-CTS-hands”) and 21 hands that were asymptomatic (DM-hands). In cases when both hands were involved, both hands were used separately for data analysis. Twenty healthy volunteers (40 hands) who had no clinical or NCS evidence of CTS and no other neurologic disorders were enrolled as normal control (“C-hands”).

The diagnostic criteria of CTS were according to the American Academy of Neurology, which included clinical history, symptoms, and evidence of slowing of distal median nerve conduction [3,10]. The clinical diagnosis of CTS was based on signs and symptoms of median nerve distribution such as 1) paresthesia, pain, swelling, weakness, or clumsiness of the hand provoked or worsened by sleep, sustained hand or arm position, or repetitive action of the hand or wrist that is mitigated by a change in posture or by shaking of the hand; 2) sensory deficits in the median nerve-innervated regions of the hand; 3) motor deficit or hypotrophy of the median nerve-innervated thenar muscles; and 4) positive provocative clinical tests (positive Phalen’s maneuver and/or Tinel’s sign). The clinical diagnosis of CTS was made when criterion 1 and one or more of criteria 2–4 were fulfilled [10,11].

Diabetes mellitus (DM) was diagnosed according to the criteria of the World Health Organization as follows: 1) fasting plasma glucose >126 mg/dl (7.0 mmol/l) where fasting was defined as “no caloric intake for at least 8 hours”; 2) symptoms of hyperglycemia and random plasma glucose >200 mg/dl (11.1 mmol/l), where random was defined as any time of day without regard to time since last meal; or 3) 2-h plasma glucose >200 mg/dl (11.1 mmol/l) during an oral glucose tolerance test [12].

### Exclusion criteria

Diabetic patients with clinical and electrophysiologic diagnosis of diabetic polyneuropathy were excluded. Clinical diagnosis was based on the recommendations of the American Academy of Electrodiagnostic Medicine (AAEM) [13]. An electrodiagnostic abnormality plus at least one sign and one symptom confirmed the presence of polyneuropathy. The neuropathic symptoms included sensory symptoms (e.g., distal numbness, burning, prickling paresthesia, dysesthesia, and allodynia) and/or motor symptoms (i.e., decreased sensibility on the distal lower extremity, distal muscle weakness or atrophy). Neuropathic signs included absent or decreased ankle deep tendon reflex, decreased or absent distal sensory capacity, distal weakness, and muscle atrophy. Abnormal electrodiagnostic studies included a sural or peroneal and one median or ulnar nerve dysfunction. However, entrapment lesions were excluded. Patients with prior surgery for CTS, and those with gout, rheumatoid arthritis, or abnormal thyroid function related to peripheral neuropathy were also excluded.

The hospital’s Institutional Review Committee on Human Research approved the study protocol and all of the participants provided informed consent. A neurologist (Dr. Cheng-Hsien Lu) experienced in NCS interpretation and another neurologist (Dr. Shu-Fang Chen) experienced in ultrasound (US) examinations evaluated the study participants. Both were blind to the status of the patients. The musculoskeletal US and NCS examinations were done by standard laboratory methods [14,15].

### Electrodiagnostic testing

The NCS was performed on all participants according to the recommended AAEM protocol using a Nicolet Viking Select system (Nicolet Biomedical Inc. Madison, USA) [2]. All tests were done under similar temperature conditions in the same room. Skin temperature was maintained at ≥32°C. The routine NCS of the upper extremities was performed on each participant. The latency, amplitude, distance, and velocity of the median and ulnar motor and sensory nerves were measured.

The motor median NCS was performed using standard techniques of supra-maximal stimulation. The distal motor nerve latency (DML) was measured with an active electrode placed over the muscle belly of the abductor pollicis brevis. The nerve was stimulated using bipolar stimulation electrodes, with the cathode positioned 2 cm proximal to the wrist crease. The cathode was placed closest to the recording electrode. The average anti-dromic median and ulnar sensory nerve action potential (SNAP) response over digit 4 was recorded using ring electrodes.

Comparative tests included median-ulnar sensory conduction between the wrist and ring finger, and median sensory nerve conduction comparison between the wrist and palm. Electrophysiologic test results considered supportive of CTS were median nerve distal sensory latency >3.4 ms; median nerve distal motor latency over the thenar >4.2 ms [16]; and difference between median and ulnar nerve distal sensory latencies >0.4 ms [17].

### Ultrasonography assessment

Ultrasonography was performed using a scanner with a 12/5-MHz linear array transducer for carpal tunnel study (Philips HDI 5000; Philips Medical Systems, Bothell, WA). During the examination, the patient sat in a comfortable position facing the examiner, with the measured forearm resting on the table, the palm supine, and fingers semi-extended in the neutral position. The transducer was placed directly on the patient’s skin with gel. For the longitudinal scan of the median nerve, the probe was placed at the midline with the center of the probe at the distal wrist crease. This provided an initial general overview of the median nerve. For the transverse scan, the probe was kept directly perpendicular to the long axis of the median nerve in order to ensure that the area measured reflected a cross-sectional area (CSA) and performed to record the CSA (direct tracing with electronic calipers around the margin of the nerve excluding the hyper-echoic epineurial rim).

The CSA and flattening ratio (FR, defined as the ratio of the major axis of the median nerve to its minor axis) were measured at the outlet of the carpal tunnel (CSA_D and FR_D, respectively) and wrist crease (CSA_W and FR_W, respectively). The “tunnel inlet” referred to the level immediately deep to the proximal edge of the flexor retinaculum. The “tunnel outlet” referred to the level immediately deep to the distal edge of the flexor retinaculum.

### Statistical analysis

Data were presented as mean ± standard deviation (SD) and compared among the groups. Continuous variables between the two groups were performed using the Student *t* test. One-way ANOVA analysis followed by Scheffe’s post-hoc comparison was used to calculate the parameters of ultrasonography and electrodiagnostic testing values, while chi-square test or Fisher’s exact test were used as appropriate to compare proportions among the groups. Spearman’s rank test was used to assess correlations between quantitative variables without normal distribution.

Receiver operating characteristics (ROC) curve analysis was used to determine the CSA value most predictive of CTS hands. Stepwise logistic regression analysis was used to identify independent predictors of ultrasonography for CTS, with adjustments made for other potential confounding factors. Statistical analysis was performed using the SPSS statistical software for Windows version 13 (SPSS, Inc., Chicago, Ill., USA). A *p* < 0.05 was considered statistically significant.

## Results

### Baseline characteristics among the groups

The demographic data of the study participants were shown in Table 1. The 80 non-DM and 40 DM patients had 81 and 59 CTS-hands, respectively. Body weight, body mass index (BMI), and wrist circumference were significantly higher in the DM patients than in the non-DM patients and controls (*p* < 0.05). There was no significant difference in terms of age, sex, and body height among the three groups.

**Table 1:** Baseline characteristics of the study participants

	**Non-DM**	**DM**	**Control**	***p *value**
	**(n = 80)**	**(n = 40)**	**(n = 20)**	
CTS hands n (%)	81 (50.6%)	59 (73.8%)	-	
Female n (%)	48 (60%)	24 (60%)	12 (60%)	0.99
Age (y)	59.3 ± 8.6	61.6 ± 8.4	54.8 ± 9.3	0.06
Body height (cm)	157.0 ± 8.0	158.2 ± 8.0	161.4 ± 8.9	0.16
Body weight (Kg)	60.6 ± 8.7	68.8 ± 16.0	62.0 ± 9.1	0.02
Body mass index	24.6 ± 3.1	27.3 ± 5.0	23.8 ± 3.4	0.01
Circumference of the wrist (mm)	160.7 ± 10.7	168.1 ± 13.7	158.3 ± 9.8	0.03

P value was calculated by one-way ANOVA analysis among the groups.

### Comparison of ultrasonographic parameters among groups

Parameters of ultrasonography among C-hands, CTS-hands, DM-CTS-hands, and DM-hands were shown in Table 2. The CSA_D and CSA_W were significantly different among the four groups (*p* < 0.01). By one-way ANOVA with Scheffe’s post-hoc comparison, CSA_D and CSA_W were significantly larger in CTS-hands and DM-CTS-hands compared to C-hands or DM-hands (*p* < 0.01). However, there is no difference of CSA_D and CSA_W between DM and non-DM CTS patients. The FR_D and FR_W were not statistically different among the four groups.

**Table 2:** Parameters of ultrasonography among the groups

	**C-hands**	**CTS-hands**	**DM-CTS-hands**	**DM-hands**	
	**(n=40)**	**(n=81)**	**(n=59)**	**(n=21)**	***p *value**
Median nerve					
CSA_D (mm^2^)	12.0±2.9	15.4±3.8	15.4±4.8	13.8±4.4	0.002^α^
Flattening ratio_D	3.7±0.9	3.6±1.0	3.5±0.7	3.7±1.2	0.39
CSA_W (mm^2^)	11.8±2.0	15.3±3.7	15.5±4.8	12.2±3.4	<0.001^β^
Flattening ratio_W	3.0±0.6	3.4±0.8	3.0±0.7	3.3±0.8	0.10

Abbreviations: *CSA* cross section area, *D* at outlet of carpal tunnel, *W* at the wrist crease.

*P* value was calculated by one-way ANOVA with post-hoc comparison:

α = CTS-hands vs. C-hands, p = 0.007; DM-CTS-hands vs. C-hands, p = 0.012.

β = CTS-hands vs. C-hands, p < 0.001; DM-CTS-hands vs. C-hands, p < 0.001; CTS-hands vs. DM-hands, p = 0.019; DM-CTS-hands vs. DM-hands, p = 0.014.

### Comparison of parameters of electrodiagnostic tests between CTS and DM-CTS groups

A summary of electrodiagnostic parameters between CTS and DM-CTS groups (Table 3) showed significant differences in terms of amplitude and motor nerve conduction velocity (NCV) of median compound motor action potential (CMAP) (*p* < 0.001). There were also significant differences in distal sensory latency, amplitude, and sensory NCV (when stimulating the middle palms [MP] and the wrist [W]) of the median SNAP between the two groups (*p* < 0.05). Latency of the median F wave was significantly longer in DM-CTS-hands compared to CTS-hands (*p* < 0.01).

**Table 3:** Parameters of electrodiagnostic testing between CTS and DM-CTS groups

	**CTS-hands**	**DM-CTS-hands**	
	**(n = 81)**	**(n = 59)**	***p *value**
Median nerve			
DML (ms)	5.2 ± 1.3	5.3 ± 1.2	0.35
CMAP (mV)	8.5 ± 2.8	6.8 ± 2.7	<0.001
MNCV (m/s)	54.6 ± 4.8	49.2 ± 5.5	<0.001
DSL (ms)	3.4 ± 0.7	3.8 ± 1.0	0.014
SNAP (μV)	25.5 ± 15.1	17.3 ± 12.7	0.001
SNCV_MP (m/s)	62.1 ± 10.4	55.9 ± 12.9	0.003
SNCV_W (m/s)	42.1 ± 7.8	38.7 ± 9.1	0.024
SNCV_D (m/s)	20.0 ± 10.2	17.2 ± 5.6	0.116
FW (ms)	27.0 ± 3.5	28.9 ± 3.5	0.003

Abbreviations: *NCV* nerve conduction velocity, *DML* distal motor latency of median CMAP, *CMAP* amplitude of median CMAP, *MNCV* motor NCV of median CMAP, *DSL* distal sensory latency of median SNAP, *SNAP* amplitude of median SNAP, *SNCV_MP* sensory NCV when stimulating at the middle palms, *SNCV_W* sensory NCV when stimulating at the wrist, *SNCV_D*, velocity difference (SNCV_MP - SNCV_W) of SNCV, *FW* median F wave. *p* value was calculated by independent *t*-test between two groups.

### The cut-off value CSA for prediction of CTS

**Figure 1:**
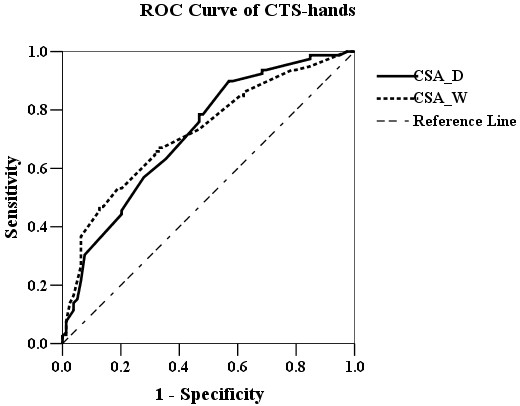
The ROC curve analysis of CSA_D (black line) and CSA_W (dot line) for CTS in non-DM patients.

**Figure 2:**
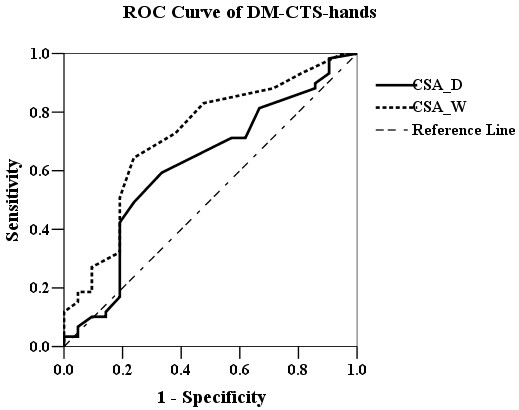
The ROC curve analysis of CSA_D (black line) and CSA_W (dot line) for CTS in DM patients.

**Figure 3:**
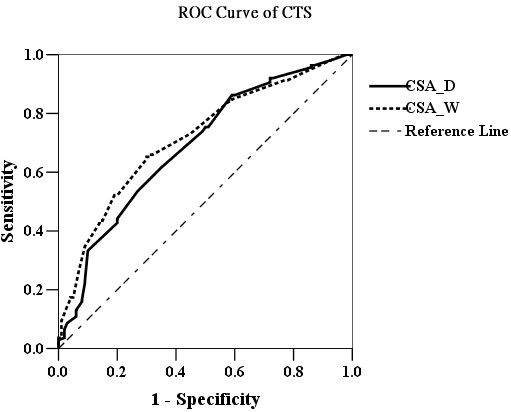
The ROC curve analysis of CSA_D (black line) and CSA_W (dot line) for CTS in all participants.

The ROC curve analysis (Figure 1) revealed that CSA_D ≥12 mm^2^ (area under curve [AUC] 0.62; sensitivity 78.5% and specificity 53.2%) and CSA_W ≥13 mm^2^ (AUC 0.72; sensitivity 78.5% and specificity 53.2%) were the most powerful predictors of non-DM CTS-hands. The ROC curve analysis of DM-CTS-hands (Figure 2) revealed that CSA_D ≥13 mm^2^ (AUC 0.72; sensitivity 71.2% and specificity 53.9%) and CSA_W ≥13 mm^2^ (AUC 0.72; sensitivity 72.9% and specificity 61.9%) were the most powerful predictors of DM-CTS-hands. The ROC curve analysis of all CTS-hands (Figure 3) revealed that CSA_D ≥12 mm^2^ (AUC 0.69; sensitivity 75.4% and specificity 50%) and CSA_W ≥13 mm^2^ (AUC 0.72; sensitivity 73.2% and specificity 55%) were the most powerful predictors of CTS.

### Correlation among glycemic state of the patients, CSA and NCS parameters

Spearman's non-parametric correlation among CSA and electrodiagnostic parameters demonstrated positive correlations in terms of CSA_D, CSA_W, distal motor latency of median CMAP, distal sensory latency of median SNAP, and latency of median F wave (Table 4). On the other hand, CSA_D or CSA_W negatively correlated with the amplitude of the median CMAP, amplitude of the median SNAP, and sensory NCV of the median nerve. The HbA1C and duration of DM were not correlated to the CSA of the median nerve.

**Table 4:** Correlation among glycemic state of the patients, CSA and electrodiagnostic testing of the median nerve

	**CSA_D**		**CSA_W**	
**Spearman’s**	***r***	***p***	***r***	***p***
DML	0.304	<0.001**	0.379	<0.001**
CMAP	-0.132	0.043*	-0.265	<0.001**
MNCV	-0.050	0.447	-0.115	0.077
DSL	0.212	0.001**	0.334	<0.001**
SNAP	-0.187	0.005**	-0.311	<0.001**
SNCV_MP	-0.039	0.557	-0.151	0.023*
SNCV_W	-0.213	0.001**	-0.366	<0.001**
SNCV_D	0.137	0.039*	0.188	0.004**
FW	0.199	0.004**	0.158	0.022*
HbA1c	0.254	0.063	0.160	0.252
Duration of DM	0.063	0.712	0.032	0.853

*Correlation is significant at the 0.05 level (2-tailed).

**Correlation is significant at the 0.01 level (2-tailed).

Abbreviations: *CSA_D* cross-section area at outlet of carpal tunnel, *CSA_W* cross-section area at the wrist crease, *NCS* nerve conduction study, *NCV* nerve conduction velocity, *DML* distal motor latency of median CMAP, *CMAP* amplitude of median CMAP, *MNCV* motor NCV of median CMAP, *DSL* distal sensory latency of median SNAP, *SNAP* amplitude of median SNAP, *SNCV_MP* sensory NCV when stimulating at the middle palms, *SNCV_W* sensory NCV when stimulating at the wrist, *SNCV_D* velocity difference (SNCV_MP - SNCV_W) of SNCV, *FW* median F wave.

### Predictive factors for CTS in DM patients

Possible predictive factors of CTS in DM participants were listed in Table 5. The BMI and CSA_W were significantly different between CTS hands and non-CTS hands in DM patients. Variables used in the stepwise logistic regression model included sex, body weight, BMI, CSA_D and CSA_W. After analysis, only CSA_W (OR 1.21, 95% CI 1.07-1.38; *p* = 0.003) was independently associated with CTS in DM patients, and any 1 mm^2^ increase in CSA_W increased the predictive rate of CTS by 28%.

**Table 5:** Predictive factors for CTS in DM patients

	**DM-CTS-hands**	**DM-hands**	***p *value**
	**(n = 59)**	**(n = 21)**	
Age (y)	61.6 ± 9.0	61.6 ± 6.5	0.99
Body height (cm)	158.7 ± 7.8	156.7 ± 8.4	0.45
Body weight (kg)	71.6 ± 17.2	61.3 ± 9.4	0.06
Body mass index	28.3 ± 5.5	24.8 ± 1.8	0.04
Circumference of the wrist (mm)	168.9 ± 13.9	166.7 ± 14.4	0.67
Flattening ratio of the wrist	2.0 ± 0.2	2.1 ± 0.2	0.51
Ultrasonography of median nerve			
CSA_D (mm^2^)	15.4 ± 4.8	13.8 ± 4.4	0.20
Flattening ratio_D	3.5 ± 0.7	3.7 ± 1.2	0.48
CSA_W (mm^2^)	15.5 ± 4.8	12.2 ± 3.4	0.006
Flattening ratio_W	3.0 ± 0.7	3.3 ± 0.8	0.22

Abbreviations: *CSA_D* cross-sectional area at the outlet of the carpal tunnel, *CSA_W* cross-section area at the wrist crease.

## Discussion

The present study has several major findings. First, the CSA of the median nerve at the outlet and wrist crease are significantly larger in CTS hands in both DM and non-DM patients compared to control hands. Second, the cut-off value of CSA at the wrist for CTS confirmation is more than 13 mm^2^ in both DM and non-DM CTS patients. Third, the CSA of the median nerve at the wrist crease is a predictive factor for CTS in DM patients and any increase of 1 mm^2^ in the CSA at the wrist crease increases the predictive rate by 28%. Lastly, the CSA of the median nerve positively correlates to the distal motor latency of the median CMAP, distal sensory latency of the median SNAP, and latency of the median F wave. On the other hand, the CSA of the median nerve negatively correlates to the amplitude of the median CMAP, amplitude of the median SNAP, and sensory NCV of the median nerve.

Significant differences in CSA measurements of the median nerve between CTS patients and asymptomatic controls have been shown in previous studies [4,14], as well as in the present study. To date, this is the first study to evaluate the value of ultrasonography between DM and non-DM patients with clinical and electrodiagnostic-confirmed CTS. The CSA of the median nerve is larger in both DM CTS and non-DM CTS groups compared to those without CTS. However, there is no difference in the CSA of the median nerve at the wrist crease or tunnel outlet between DM and non-DM CTS patients.

Several studies have explained the phenomenon of local enlargement of the median nerve in CTS, including nerve constriction at the site of the entrapment with proximal swelling [18], and the presence of Renaut bodies [19]. The biological response to compression seems to be a cascade composed of endoneurial edema, demyelination, inflammation, distal axonal degeneration, fibrosis, growth of new axons, re-myelination, and thickening of the perineurium and endothelium [20,21]. A recent study has demonstrated focal enlargement of median nerve CSAs in diabetic patients, especially at the level of the inlet. Other additional factors may contribute to the phenomenon, including a reduction in myelinated nerve fibers and capillary density that may predispose DM patients to develop CTS [22], and the polyol pathway, glycation and pro-inflammatory reactions that are known to contribute to diabetic peripheral nerve injuries [23]. The reasons why CSAs between DM and non-DM groups are not significant in the present study may be that the biological response to compression is a more important contributing factor than diabetic peripheral nerve injuries.

Previous reports show that increased CSA of the median nerve is the most predictive parameter for non-DM CTS [24]. The reported critical values for CSA vary between 9 and 15 mm^2^ in the non-DM group. The cause of this variability may be the differences in study design, race, grading severity, and measurement techniques [7,25-29]. However, most of these studies exclude DM patients to eliminate possible confounding factors of diabetic peripheral neuropathy (DPN) in diagnosing CTS. A previous study has demonstrated that the CSA of the median nerve in the carpal tunnel of DPN patients is greater than that of non-DPN patients [30]. However, their study did not exclude asymptomatic CTS in diabetic patients.

The present study excluded diabetic patients with DPN (e.g. polyneuropathy or mononeuropathy multiplex) by NCS and clinical presentations in order to focus on the entrapment effects of CTS. The cut-off value of CSA at the wrist crease for CTS confirmation is more than 13 mm^2^ in both DM and non-DM CTS patients. There is no statistical difference in the median CSA between DM CTS and idiopathic CTS patients in this study. The results suggest that the entrapment factor may drown out other factors like metabolic and vascular causes of median nerve enlargement in CTS.

Median nerve swelling as detected by calculating the CSA reflects the degree of nerve damage expressed by the clinical picture. The CSA of the median nerve has a diagnostic value for confirming or excluding carpal tunnel syndrome. However, the relationship among the CSA of the median nerve, severity of nerve conduction study (NCS), and clinical severity remains controversial [4,29,31]. In this study, the CSA of the median nerve positively correlates with distal motor latency of the median CMAP and distal sensory latency of the median SNAP. In other words, suspicious enlargement of the median nerve damages the myelin and results in the slowing of nerve conduction velocity. On the other hand, the CSA of the median nerve negatively correlates with the amplitude of the median CMAP and median SNAP, suggesting that median nerve swelling contributes to axonal degeneration of the median nerve in CTS. Thus, larger CSA points to more median nerve damage.

Although electrodiagnostic studies are highly specific, false negativity can be seen in the variable ratio of 10-20% [25,32]. Nerve conduction studies mainly involve large nerve fibers and only identify permanent nerve damage, which is why the NCS is sometimes negative. Although median nerve function remains intact, the nerve can already be increasing in size at the carpal tunnel inlet, as shown by ultrasonography. The consensus of electrodiagnostic study for CTS is not specific for DM patients [2,16,17]. The diagnostic criteria are dependent on the distal latency and NCV of the median nerve as the constellation of axonal degeneration is manifested in reduced CMAP amplitude or underestimated SNAP. The results here demonstrate that the CSA of the median nerve at the wrist crease is independently associated with CTS in DM patients and that any 1 mm^2^ increase in CSA will increase the predictive rate of CTS by 28%. As such, ultrasonography may be considered an alternative diagnostic modality when NCS results are not confirmatory in patients suspected of CTS. The combination of NCS and US may be able to estimate CTS with diabetes mellitus more accurately.

This study has several limitations. First, the electrodiagnostic unit is in a tertiary referral center with various specialties like general medicine, orthopedics, and neurology, while the study group is limited to patients with CTS as a primary diagnosis. A selection bias is inherent in this type of hospital-based study and the results may not be applicable to primary care. Second, ultrasonography is an operator-dependent test and appropriate experience is required to ensure reliability and reproducibility. With two experienced physicians performing the US examination, this issue is easily resolved. Third, DM patients with suspected polyneuropathy or mononeuropathy multiplex by clinical presentation and NCS were excluded, which may cause potential bias in statistical analysis. Fourth, the case number is small, with multiple confounding factors, especially in the DM group. Finally, the association between symptom duration and US findings is an interesting finding. However, symptoms in most CTS patients here have an insidious onset, the exact duration of symptoms can not be determined for correlation with the severity of US data.

## Conclusions

In conclusion, the CSA of the median nerve by ultrasonography can be a diagnostic modality for evaluating CTS. Median nerve swelling is detected by calculating the CSA, which reflects demyelination and axonal degeneration of the median nerve. The cut-off value of CSA at the wrist for CTS confirmation is 13 mm^2^ for both DM and non-DM patients. The CSA of the median nerve at the wrist crease is a predictive factor for CTS in diabetic patients.

### Ethical approval

Chang Gung Memorial Hospital’s Institutional Review Committee on Human Research approved the study (IRB100-1390B and IRB100-2862C).

## Abbreviations

AAEM: American Academy of Electrodiagnostic Medicine; AUC: Area under curve; BMI: Body mass index; CTS: Carpal tunnel syndrome; CMAP: Compound motor action potential; CSA: Cross-sectional area; DM: Diabetes mellitus; DML: Distal motor nerve latency; EMG: Electromyography; FR: Flattening ratio; NCS: Nerve conduction studies; NCV: Nerve conduction velocity; ROC: Receiver operating characteristics; SD: Standard deviation; SNAP: Sensory nerve action potential; US: Ultrasound

## Competing interests

The authors declare that they have no competing interests.

## Pre-publication history

The pre-publication history for this paper can be accessed here:

http://www.biomedcentral.com/1471-2377/13/65/prepub
